# Medium Chain Acyl‐CoA Dehydrogenase Deficiency; an Unexpected Cause of Neonatal Ketoacidosis

**DOI:** 10.1002/jmd2.70118

**Published:** 2026-08-10

**Authors:** Nazreen Kamarus Jaman, Isaac Bernhardt, Sophie Ward, Freya Hassall, James Nurse, Hugh Lemonde, R. Neil Dalton, Michael Champion

**Affiliations:** ^1^ Department of Inherited Metabolic Disease Evelina Children's Hospital, Guys & St Thomas' NHS Foundation Trust London UK; ^2^ Biochemical Sciences Viapath, Guys & St Thomas' NHS Foundation Trust London UK; ^3^ Department of Paediatrics University Hospital, Southampton NHS Foundation Trust Southampton UK; ^4^ WellChild Laboratory Evelina London Children's Hospital, Guys & St Thomas' NHS Foundation Trust London UK

**Keywords:** cardiomyopathy, hypoglycaemia, MCAD, neonatal ketoacidosis

## Abstract

Medium‐chain acyl‐CoA dehydrogenase deficiency (MCADD) classically presents with hypoketotic hypoglycaemia; however, this presentation is now rare following the introduction of newborn screening. While children with MCADD may produce some ketones, severe ketoacidosis has not been previously described. Here we report two patients with MCADD presenting with severe ketoacidosis in the neonatal period prior to results of newborn screening. Patient 1 presented on Day 5 with hypoglycaemia, profound ketoacidosis and circulatory shock, and developed refractory ventricular tachycardia requiring extra‐corporeal membrane oxygenation. Patient 2 presented on Day 4 with severe ketoacidosis, but only borderline hypoglycaemia. The diagnosis of MCADD was rapidly confirmed in both by analysis of acylcarnitines and urine organic acids, with subsequent genetic confirmation of *ACADM* mutations. We conclude that MCADD should be included in the differential diagnosis of neonatal ketoacidosis, with or without hypoglycaemia.

## Synopsis

MCAD deficiency may present with severe neonatal ketoacidosis.

## 1 Introduction

Medium‐chain acyl‐CoA dehydrogenase deficiency (MCADD, OMIM#201450) affects approximately 1/10 000 newborns in the United Kingdom. It occurs due to biallelic pathogenic variants in ACADM, leading to impaired β‐oxidation of medium chain acyl‐CoAs [1, 2]. This results in impaired ability to generate energy and acetyl‐CoA from medium‐chain fatty acids, with corresponding accumulation of medium‐chain acylcarnitines. Acetyl‐CoA is required for ketone synthesis, and the classical clinical presentation of MCADD is with acute encephalopathy associated with hypoketotic hypoglycaemia, precipitated by inadequate energy intake during early childhood illnesses. This presentation is now rare following the widespread inclusion of MCADD in newborn screening programmes, which in the United Kingdom is undertaken on Day 5 by electrospray tandem mass spectrometry in dried bloodspots [2]. However there is a well‐described risk of neonatal decompensation prior to the newborn screening result, especially in breastfed babies, which may be fatal [3, 4].

It is also recognized that children with MCADD are able to generate some ketones while fasting [5, 6, 7]. Affected children are nevertheless assumed to have a relatively blunted ketone response, and as such, MCADD is not typically considered in the differential diagnosis of ketoacidosis. Here we present two patients with neonatal presentations of MCADD associated with severe ketoacidosis.

### 1.1 Patient 1

Patient 1 is a 15‐year‐old male who initially presented on Day 5 of life after a normal pregnancy and birth. He was exclusively breastfed from birth and was taken to the emergency department due to poor feeding on Day 4, but was discharged with lactation consultant input. However he became increasingly lethargic, and an ambulance was called on Day 5. With the ambulance crew he had a blood glucose of 1.7 mmol/L, treated initially with glucagon. On arrival in hospital he was maintaining his oxygen saturations in 15 L but with poor perfusion. Initial point of care testing revealed a severe metabolic acidosis with persisting hypoglycaemia (2.2 mmol/L) and normal lactate (Table 1).

**TABLE 1:** Biochemical parameters at the time of initial acute presentation, biochemical genetics results and genotype for P1 and P2.

Results (reference range)	Patient 1	Patient 2
Glucose (> 2.7)	1.7 mmol/L	2.6 mmol/L
pH (7.35–7.45)	6.9	7.1
Base excess (−5 − +5)	−31 mmol/L	−23 mmol/L
Anion gap (< 20)	16 mmol/L	26 mmol/L
Lactate (< 2.2)	1.3 mmol/l	1.6 mmol/L
Ketones (< 0.4)^a^	N/A	6.5 mmol/L
Ammonia (< 70)	140 μmol/L	123 μmol/L
Alanine transaminase (< 55)	28 U/L	110 U/L
Creatine kinase (< 220)	674 IU/L	1662 IU/L
Acylcarnitine profile (plasma)	N/A	C8 = 7.78 μmol/L (at presentation Day 4)
Acylcarnitine profile (DBS)		
C0 (15–45)	C0 = 4.3 μmol/L	C0 = 12.0 μmol/L
C8 (< 0.2)	C8 = 8.4 μmol/L	C8 = 2.4 μmol/L
C8/C10 (< 1.0)	C8/C10 ratio = 6.2 (at presentation Day 5)	C8/C10 = 7.3 (at presentation Day 4)
Newborn screening sample (DBS—collected on Day 5, C8 cut‐off < 1.0)^b^	C8 = 3.4 μmol/L	C8 = 0.77 μmol/L
	C8/C10 ratio = 8.3	C8/C10 = 8.9
Urine organic acids	Gross ketonuria, heavy dicarboxylic aciduria, moderately large suberylglycine peak, trace of hexanoylglycine	Marked ketonuria and dicarboxylic aciduria, increased excretion of hexanoylglycine and suberylglycine
	(at presentation Day 5)	(at presentation Day 4)
*ACADM* genotype	Homozygous c.985G>A p.(Lys329Glu)	c.985A>G p.(Lys329Glu); c.1055A>G p.(Tyr352Cys)

Abbreviations: C0: free carnitine; C8: octanoylcarnitine; C10: decanoylcarnitine; DBS; dried bloodspot; N/A; not available.

^a^ Blood ketones measured by point of care ketone meter. Note that blood ketones were not measured at the time of initial presentation in P1.

^b^ Note that newborn screening DBS samples are routinely collected on Day 5 in the United Kingdom.

He was treated with intravenous glucose and was intubated and ventilated, and transferred to the regional Paediatric Intensive Care Unit (PICU). ST segment depression was noted on cardiac monitoring with anteroseptal dyskinesia on echocardiogram, and he then developed frequent runs of ventricular tachycardia that were refractory to treatment. Left‐ventricular function continued to deteriorate, necessitating emergency initiation of extra‐corporeal membrane oxygenation (ECMO).

In view of the profound acidosis (pH 6.9) with hyperammonaemia (140 μmol/L), an organic acidaemia was suspected and he was commenced on levocarnitine and intralipid on Day 6 of age (Table 1). Urine for organic acids was collected on Day 5 and reported on Day 7, showing profound ketonuria and heavy dicarboxylic aciduria, with a moderately large peak of suberylglycine and a trace of hexanoylglycine. Blood or urine ketones were not measured at the time of initial presentation, and therefore the profound ketoacidosis was not appreciated before this result. The degree of ketosis was consistent with a disorder of ketone utilisation; however there was no increase in 2‐methylacetoacetate, tiglylglycine or 2‐methyl‐3‐hydroxybutyrate, making β‐ketothiolase deficiency unlikely. Succinyl‐CoA:3‐ketoacid CoA transferase deficiency was proposed as a possible explanation. The presence of elevated suberylglycine and hexanoylglycine was considered to be consistent with excessive fatty acid oxidation and/or MCADD carrier status. Subsequent testing for activity of succinyl‐CoA:3‐ketoacid CoA transferase and mitochondrial acetoacetyl‐CoA thiolase in fibroblasts was normal.

Acylcarnitines, which were performed in dried bloodspots by electrospray tandem mass spectrometry on Day 7 by a reference laboratory, showed markedly increased octanoylcarnitine (8.4 μmol/L) indicating a likely diagnosis of MCADD (Table 1). Newborn screening results returned subsequently and were also consistent with MCADD, which was confirmed following the detection of homozygosity for the common pathogenic *ACADM* variant c.985A>G, p.(Lys329Glu).

ECMO was successfully discontinued after 3 days, and regular feeds were established with breastmilk and formula. Ketonuria resolved by Day 9. Cardiac function was normal thereafter, and he had no further episodes of metabolic decompensation or ketoacidosis, but developed a tic disorder and seizures which were controlled on levetiracetam. He is now 15 years of age, has a diagnosis of autism spectrum disorder, and attends a mainstream school.

### 1.2 Patient 2

Patient 2 presented on Day 4, with a history of intermittent noisy breathing since birth. He was reported to be breastfeeding well initially; however feeds became shorter and more frequent overnight on Day 3. On Day 4 he became suddenly floppy and poorly responsive, and was found to be pale, hypotensive and hypothermic with grunting respiration. Initial blood gas revealed borderline hypoglycaemia with severe ketoacidosis and normal lactate (Table 1). Following fluid resuscitation, intubation, intravenous antimicrobials and glucose infusion, he was retrieved to the PICU at the metabolic referral centre.

Acylcarnitines were analysed in underivatized plasma by flow injection electrospray tandem mass spectrometry when the patient was first admitted to the metabolic referral centre. Results revealed a marked elevation of octanoylcarnitine (7.78 μmol/L), suggesting a diagnosis of MCADD. Newborn screening resulted on Day 7 of life, was also consistent with MCADD as were subsequent dried bloodspot acylcarnitines, but with milder elevations of octanoylcarnitine following further intravenous glucose (Table 1). Urine organic acids showed marked ketonuria and increased hexanoylglycine and suberylglycine consistent with MCADD. Molecular analysis confirmed the diagnosis, with extended Sanger sequence analysis of the *ACADM* gene showing apparently compound‐heterozygous *ACADM* variants; c.985A>G p.(Lys329Glu) and the c.1055A>G p.(Tyr352Cys) likely pathogenic variant.

The acidosis improved rapidly with intravenous glucose therapy, and P2 was successfully extubated the day after presentation, with no neurological deficits apparent on examination. Mixed breast and bottle feeding was established prior to discharge, and serial pre‐feed blood glucose and ketone levels remained normal. He remains well at 4 months of age with no further decompensations and no concerns regarding growth or development.

## 2 Discussion

This report describes two newborns with MCADD presenting with severe ketoacidosis, broadening the phenotypic spectrum of MCADD, and the differential diagnosis for neonatal ketoacidosis. Although it is well‐known that the presence of ketones does not exclude a diagnosis of MCADD, presentation with severe ketoacidosis is seemingly paradoxical, and to our knowledge has not previously been reported. Acidosis was not due to hyperlactataemia, which may occur during acute MCADD presentations due to tissue hypoperfusion and cellular energy failure. Disorders of ketone body utilization or transport were suspected initially in both patients, with organic acidaemias also considered in the differential diagnosis (Table 2). Despite the biochemical diagnosis of MCADD in P1 it was nevertheless considered prudent to exclude a concomitant ketolysis defect. Further investigations were not considered necessary following the rapid confirmation of MCADD in P2, in view of our prior experience.

**TABLE 2:** Differential diagnosis for neonatal ketoacidosis.

	Disorder		Key additional features
Hyperglycaemia	Neonatal diabetes mellitus^a^		Dehydration
			Anion gap ↑
Normoglycaemia or hypoglycaemia^a^	Inherited metabolic disorders	Organic acidaemias	Encephalopathy
			Hyperammonaemia ++
			Anion gap ↑↑↑
			Dehydration
		Disorders of ketone utilisation or transport	Permanent or fasting ketosis
			Anion gap ↑↑
		Fatty acid oxidation disorders (usually hypoketotic; ketoacidosis rare)	Encephalopathy Acute cardiomyopathy Elevated creatine kinase and transaminases Hyperammonaemia +/−
		Maple syrup urine disease	Encephalopathy
			Ketonuria (acidosis rare)
		Others including defects of respiratory chain, gluconeogenesis, multiple carboxylase	Lactic acidosis usually more prominent than ketosis
	Others; starvation ketosis, sepsis		Significant ketosis unusual
			Consider underlying IMD

^a^ Ketoacidosis with transient hyperglycaemia may occur in sepsis, organic acidaemias and ketolysis defects, which may initially resemble diabetic ketoacidosis.

In contrast to the long‐chain fatty acid oxidation disorders (LC‐FAOD), patients with MCADD are able to produce some ketones from partial β‐oxidation of long‐chain acylcarnitines to medium‐chain acylcarnitines [5, 6, 7]. However it should also be noted that significant ketonuria has been seen in a patient with very long‐chain acyl‐CoA dehydrogenase deficiency, as previously reported by our unit [8]. P1 was in extremis at the time of presentation, with hypoglycaemia reflecting exhaustion of energy stores, and severe cardiac dysfunction. Acute ventricular dysfunction and arrhythmia are reported in MCADD [9, 10], but are not typically as frequent or severe as seen in the classical LC‐FAODs, with requirement for ECMO only reported once previously [10].

By contrast, P2 presented with only mild hypoglycaemia and no evidence of cardiac dysfunction, yet had marked hyperketonaemia in the range observed in diabetic ketoacidosis and ketolysis defects. The presenting blood glucose in P2 would not be universally considered abnormal in the neonatal setting, and the phenotypic spectrum of MCADD may therefore be expanded to include ketoacidosis in the absence of significant hypoglycaemia. This underscores the fact that hypoglycaemia is an unreliable indicator of metabolic decompensation in MCADD. Ketoacidosis in these profoundly catabolic infants is likely to reflect overwhelming mobilisation and partial oxidation of long‐chain fatty acids; however the severity of ketosis is difficult to explain. It is possible that ketone production from the ketogenic amino acids leucine and lysine was increased, but these are generally thought to be a minor contributor to ketogenesis, and plasma amino acid profiles in both patients were largely unremarkable.

It is noteworthy that these patients presented clinically on Day 4, before the time of routine newborn screening which in the United Kingdom occurs on Day 5, later than many programmes internationally. Neonatal decompensation and mortality can occur within the first 2 days of life in newborns with MCADD [3, 4], and such cases are not likely to be preventable even with very early newborn screening sample collection. However early NBS may enable expedited treatment or prevention of decompensation occurring at Days 4–5 in some newborn screening programmes, which might improve outcomes.

It is a potential limitation of this report that broad genomic sequencing was not performed in either patient. This is increasingly routine in some centres and could arguably be justified to exclude additional genetic pathology contributing to the phenotype, but was not considered necessary for clinical care in either patient.

In conclusion, these cases emphasize the importance of considering inherited metabolic disorders early in the evaluation of unwell neonates. Measurement of ketones should be included in the initial evaluation, and the finding of ketosis in a newborn should prompt consideration of inherited metabolic disorders including fatty acid oxidation disorders. Although included in most newborn screening programs, MCADD may present clinically before the sample is taken or reporting of results and should be considered in the differential diagnosis of neonatal ketoacidosis.

## Author Contributions

Nazreen Kamarus Jaman and Isaac Bernhardt contributed equally to this work; they both contributed to writing the first and subsequent drafts, collected data and provided clinical care. Sophie Ward and Freya Hassall conducted biochemical genetics analyses, collated and interpreted biochemical data and reviewed and revised the manuscript. James Nurse collected data, contributed to drafting the manuscript, provided clinical care and reviewed and revised the manuscript. Hugh Lemonde and Michael Champion conceptualized the study, provided patient clinical care, coordinated and supervised data collection and reviewed and revised the manuscript. Neil Dalton conceptualized the study, conducted biochemical genetics analyses, collated and interpreted biochemical data, coordinated and supervised data collection and reviewed and revised the manuscript. All authors approved the final manuscript as submitted and agree to be accountable for all aspects of the work.

## Funding

The authors have nothing to report.

## Ethics Statement

The authors have nothing to report.

## Consent

Written informed consent for publication was obtained for all patients.

## Conflicts of Interest

The authors declare no conflicts of interest.

## Data Availability

The data that support the findings of this study are available from the corresponding author upon reasonable request.
